# Crack Detection in Civil Infrastructure Using Autonomous Robotic Systems: A Synergistic Review of Platforms, Cognition, and Autonomous Action

**DOI:** 10.3390/s25154631

**Published:** 2025-07-26

**Authors:** Rong Dai, Rui Wang, Chang Shu, Jianming Li, Zhe Wei

**Affiliations:** School of Computer Science, Civil Aviation Flight University of China, Guanghan 618307, China; dairong@cafuc.edu.cn (R.D.); wr818@cafuc.edu.cn (R.W.); shuchang0530@163.com (C.S.); findz@cafuc.edu.cn (Z.W.)

**Keywords:** robotics, autonomous inspection crack detection, computer vision

## Abstract

Traditional manual crack inspection methods often face limitations in terms of efficiency, safety, and consistency. To overcome these issues, a new approach based on autonomous robotic systems has gained attention, combining robotics, artificial intelligence, and advanced sensing technologies. However, most existing reviews focus on individual components in isolation and fail to present a complete picture of how these systems work together. This study focuses on robotic crack detection and proposes a structured framework that connects three core modules: the physical platform (robots and sensors), the cognitive core (crack detection algorithms), and autonomous action (navigation and planning). We analyze key technologies, their interactions, and the challenges involved in real-world implementation. The aim is to provide a clear roadmap of current progress and future directions, helping researchers and engineers better understand the field and develop smart, deployable systems for infrastructure crack inspection.

## 1. Introduction

Cracks are the most direct and critical manifestation of structural aging in civil infrastructures such as bridges, tunnels, and dams. Their timely and accurate detection is vital for predictive maintenance and structural safety. Traditionally, crack inspection has relied heavily on manual visual assessments, a method plagued by subjectivity, low efficiency, and limited coverage. These limitations are further exacerbated by the inaccessibility of many key structural components (e.g., high cables, interior box girders), making manual inspection both costly and error-prone [1,2,3].

In response, robotic systems for autonomous crack detection have emerged, driven by advances in robotics, artificial intelligence, and sensor technologies. Unlike traditional tools, these systems represent a fundamental shift beyond simple automation, establishing an entirely new inspection paradigm. Robots are evolving into intelligent agents capable of executing the full perception–cognition–action loop. Modern robotic platforms such as UAVs and wall-climbing robots can access hard-to-reach areas safely and efficiently, while carrying multi-modal sensors (e.g., high-resolution cameras, LiDAR, NDT tools) to capture rich structural information [4,5,6,7]. These capabilities enable dense data acquisition, supporting higher-level applications like digital twin construction and condition-based maintenance.

Despite this being a prominent research area, existing reviews often adopt a fragmented approach, providing descriptive enumerations of individual technologies (platforms, sensors, or algorithms) in isolation. They typically lack a unified analytical framework to reveal the intrinsic, synergistic relationships among these components, leaving researchers and engineers struggling to navigate real-world design trade-offs and deployment challenges.

The contribution of this research is as follows. First, we propose an integrative “Platform–Cognition–Action” analytical framework. This framework is built on the core thesis that a fully functional autonomous inspection robot is a tightly coupled entity, and its performance stems from the synergy and trade-offs among its components. Second, within this framework, we systematically analyze the key technologies in each module and explicitly reveal their interdependencies, examining critical trade-offs such as mobility versus stability in platforms, efficiency versus precision in algorithms, and autonomy versus reliability in action. Third, we identify core challenges and future trends from a holistic, system-level viewpoint, arguing that breakthroughs will come from synergistic enhancements. Ultimately, this work provides researchers and engineers with a structured technology roadmap to better navigate the design trade-offs and practical challenges in developing autonomous field-deployable inspection systems.

**Figure 1:**
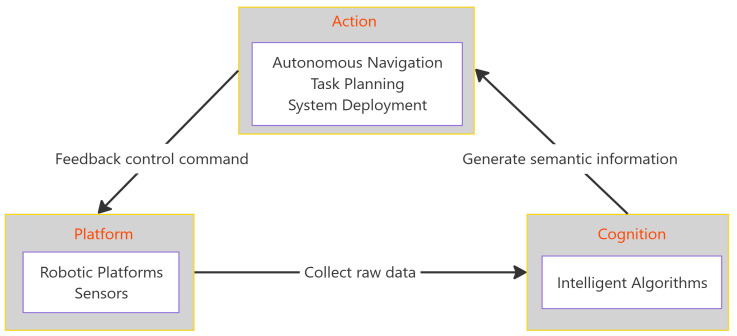
The proposed “Platform–Cognition–Action” integrative analytical framework.

As illustrated in Figure 1, the proposed framework deconstructs the robotic inspection system into three interconnected modules. The Physical Platform, reviewed in Section 2, represents the hardware (robots and sensors) that serves as the system’s interface with the physical world. The Cognitive Core, the focus of Section 3, encompasses the algorithms that process sensor data for tasks like crack detection, acting as the system’s brain. Finally, Autonomous Action, detailed in Section 4, involves the high-level planning and navigation strategies that translate cognitive understanding into purposeful movement. The framework highlights that a system’s overall effectiveness arises from the seamless integration and synergy among these modules. Building on this integrated view, Section 5 analyzes the core challenges and future technological trends, and Section 6 concludes the study.

## 2. The Physical Platform of Robotic Inspection Systems

As the bridge between the digital and physical worlds, the robotic system serves as the physical embodiment for conducting autonomous inspections. Its design is not an arbitrary selection of existing platforms but a strategic process driven by the inspection target and environmental constraints, involving trade-offs among mobility, stability, and accessibility. The core value of a fully functional physical platform lies in its ability to efficiently and safely deploy specific sensor payloads to predetermined locations on the infrastructure while providing the necessary operating conditions (such as a stable posture, precise positioning, or sufficient energy). This section follows the logic of platform design determined by sensing needs, first analyzing the sensor technologies that define the boundaries of information acquisition and, second, delving into the various robotic platforms developed to meet these sensing needs, ultimately revealing the close synergy and trade-off relationship between them.

### 2.1. Sensor Payloads

The selection and configuration of sensors mark the starting point of the entire inspection task, directly defining the dimensions and quality of information the robotic system can acquire. The field’s evolution clearly reflects a progression from relying on single visual imaging to the deep fusion of multi-modal sensing, a profound leap from merely seeing surface cracks to understanding overall structural health.

The high-resolution visual camera (RGB Camera) is the foundational payload for nearly all crack detection robots, offering an economical and efficient means of acquiring surface texture and color for image-based recognition algorithms [1,8]. However, visual information alone has significant limitations. Imaging quality is highly susceptible to environmental factors like lighting and shadows, and as 2D sensors, they cannot directly provide the depth information needed for precise spatial localization and dimensional quantification (e.g., width, depth) [9,10]. Studies show that while standards from the American Society for Testing and Materials (ASTM) like ASTM D4788-20 [11] recommend high precision, traditional cameras often fail to resolve micro-cracks (e.g., 0.3–0.4 mm wide) at typical inspection distances, necessitating a transition to 3D and fused sensing technologies [12].

To overcome the bottlenecks of vision-only systems, the fused application of multi-modal sensors has become an inevitable trend. A primary fusion strategy involves introducing 3D depth information. By tightly coupling RGB cameras with depth sensors (e.g., RGB-Depth (RGB-D) cameras), LiDAR, or structured light cameras, a robotic system can model environmental geometry with high precision, overcoming the limitations of 2D images in crack localization and quantification [4,7]. Furthermore, the deep fusion of 3D sensors with an Inertial Measurement Unit (IMU) is a key technique for enhancing perceptual robustness, particularly for achieving centimeter-level positioning and mapping in Global Navigation Satellite System (GNSS)-denied environments like tunnels [13,14].

Another key direction is integrating professional Non-Destructive Testing (NDT) sensors, transforming the robot from a surface scanner into a mobile analysis platform for detecting subsurface defects. The choice of sensor depends on the material: infrared thermography can detect concrete voids and moisture [15], while contact-based probes are needed for deeper defects. For instance, steel structures can be inspected with eddy current sensors for fatigue cracks [16] or ultrasonic probes for internal micro-defects [17]. For composite materials, acoustic sensors can be combined with Convolutional Neural Network (CNN) models for high-precision classification of interface voids [18]. Such sensors typically require physical contact with the inspected structure or maintaining a very close distance and are therefore often deployed on collaborative Unmanned Ground Vehicle (UGV)-manipulator systems [19] or have spurred the development of specialized contact-based robots (e.g., magnetic adhesion climbing robots).

Finally, the application of acoustic sensors has demonstrated unique value. Beyond NDT use (e.g., with hammer impact modules [20]), acoustic arrays have been innovatively employed to solve localization problems in GPS-denied spaces, such as inside pipelines or large metal enclosures [21], providing unique solutions for robot operations in extreme environments. Table 1 summarizes the key characteristics of these main sensor technologies.

### 2.2. Robotic Platforms

**Figure 2:**
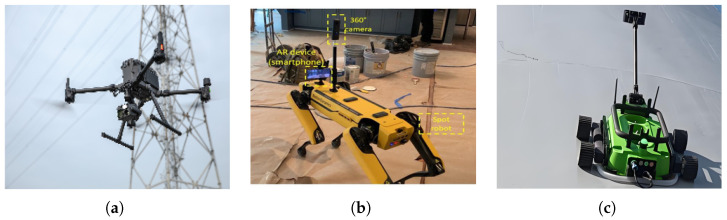
Representative robotic platforms for crack detection. (**a**) Ground mobile robot [28], (**b**) Quadruped robot [29], (**c**) Multi-rotor UAV.

Once sensing requirements are defined, the primary role of the robotic platform is to provide the necessary mobility, accessibility, and stability for the mounted sensors, thereby ensuring high-quality data acquisition. The selection and design of a platform thus becomes a process of optimizing for specific task requirements by navigating the fundamental trade-off between mobility and stability. This trade-off has led to the development of two mainstream platform categories (as shown in Figure 2), each tailored to different crack detection strategies: non-contact remote sensing and contact-based close-range inspection.

Non-contact remote sensing platforms, typified by multi-rotor Unmanned Aerial Vehicles (UAVs), are engineered for large-scale, high-efficiency inspections, aiming to rapidly conduct comprehensive crack surveys of entire structures. These platforms form a natural synergy with lightweight, non-contact sensors like visual cameras and thermal imagers. The high mobility of UAVs enables them to quickly cover large structures such as bridges and dams, capturing global overview information [12]. However, their core challenge lies in maintaining imaging stability while counteracting environmental disturbances (e.g., wind gusts) and self-induced motion during flight. Research efforts to address this are multi-faceted, combining physical platform innovations with advanced algorithmic compensation. When perfect physical stability is unattainable, a crucial direction is to perform motion compensation at the data and algorithmic levels. A primary approach involves multi-sensor fusion, where data from various sensors is integrated to build a more accurate model of the robot’s motion. For instance, recent work explicitly addresses the issue of UAV-induced motion by integrating an Inertial Measurement Unit (IMU) with laser distance sensors to capture low-frequency movements. This sensor data is then processed by an Extended Kalman Filter (EKF) to accurately estimate and correct for five of the six degrees of freedom of motion, significantly enhancing the precision of dynamic measurements [30]. Similar principles of fusing data from accelerometers and gyroscopes with Kalman filters are also applied to correct motion disturbances on other moving platforms, demonstrating the robustness of this approach [31]. Another strategy leverages computer vision techniques, using feature matching algorithms (e.g., Speeded Up Robust Features (SURF)) to calculate the homography between image frames and correct for distortions caused by UAV wobble [32]. In addition to algorithmic solutions, researchers are also developing innovative physical designs, such as tethered UAVs capable of “perching” on power lines for long-term, zero-power observation [33]. Beyond post-processing, state-of-the-art research also focuses on proactive, perception-aware motion planning controllers that anticipate and minimize motion blur during inspection maneuvers [34].

In GNSS-denied environments like tunnels and box girders, the design focus shifts toward extreme miniaturization and physical fault tolerance to enable internal crack exploration. This has led to the development of small UAVs weighing under 400 g [35] and nano-UAVs less than 10 cm in size [36], which trade payload capacity for enhanced mobility in confined spaces. Furthermore, some designs incorporate protective cages, allowing UAVs to perform exploratory inspections in dark tunnels by safely colliding and rebounding off surfaces [37].

Contact-based close-range sensing platforms sacrifice some mobility to achieve higher stability and greater payload capacity, enabling fine-grained, quantitative inspection of specific crack areas.

- Wall-climbing robots are a major research focus in this area, with their core technology revolving around reliable adhesion and movement on various vertical surfaces (e.g., steel, concrete). For steel structures, magnetic adhesion robots [38,39] have been developed in diverse forms, such as adaptive magnetic wheelsets [40], variable track chassis [41], and bionic inchworm-like structures [42], to achieve stable locomotion on complex curved surfaces and over obstacles. This combination of robust mobility and high payload capacity allows these robots to carry heavy NDT sensors, such as ultrasonic probes, and perform inspections in harsh environments like high-temperature settings [17], which is critical for detecting internal fatigue cracks at key welds. For non-ferromagnetic surfaces like concrete, negative pressure adhesion robots [4] have demonstrated significant potential. By optimizing the sealing design of their suction cavities, they can carry heavy payloads, such as a six-degrees-of-freedom robotic arm, for precise measurement of crack width and depth on concrete bridges.
- Pipe and confined-space robots are engineered to enter areas completely inaccessible to humans for internal crack inspection. To navigate the complex and variable environments inside pipes, researchers have developed various platforms, including elastic-hinged robots that passively adapt to changing pipe diameters [43], and multi-link [44] and wheeled robots [45] that can actively extend and retract. Bio-inspired snake-like robots [46] offer a unique solution for entering narrow, curved pipes to inspect inner walls for circumferential or longitudinal stress cracks. Innovations also include soft robots capable of traversing small cables with diameters under 1 mm [47], demonstrating how platform design evolves to meet sensing needs in extreme environments. However, a key technical bottleneck common to all such platforms is achieving reliable long-term autonomous localization and navigation in these signal-denied, feature-sparse environments to generate accurate internal crack maps.
- Ground robots typically possess the greatest payload capacity and endurance, often serving as mobile base stations or for inspecting ground-level cracks. They are adapted to different terrains through wheeled, legged, or hybrid (e.g., leg–wheel–track [48]) locomotion systems. In recent years, legged platforms, particularly quadruped robots, have gained significant attention for their exceptional dynamic stability and obstacle-crossing capabilities [49]. In practical applications, ground robots frequently act as hubs for heterogeneous collaborative systems. For instance, a ground robot might inspect road surface cracks on a bridge deck while simultaneously serving as a mobile base station to provide differential Global Navigation Satellite System (GNSS) signals and charging for UAVs inspecting the bridge’s towers.

Collaborative and heterogeneous platforms represent an emerging and significant trend. By combining platforms with different physical attributes, the overall crack detection capabilities of a robotic system can be substantially expanded. For example, the “quadruped robot + UAV” heterogeneous system developed by Chu et al. [37] assigns large-scale movement and global scanning in a tunnel to the ground robot, while the UAV is dispatched for local, detailed inspection of suspicious cracks in areas inaccessible to its counterpart. This model, which synergizes the high efficiency of aerial platforms with the long endurance and high payload of ground platforms, presents an effective solution for the comprehensive, in-depth crack detection of large and complex structures. Table 2 provides a detailed comparison of the key characteristics, application scenarios, and pros and cons of various platforms.

The selection of a physical platform and its sensor payload is therefore a tightly coupled engineering process. The platform’s physical limitations, such as payload, stability, and endurance, directly define the quality and type of data that can be acquired, establishing the fundamental constraints for the cognitive core algorithms to be examined next.

## 3. The Cognitive Core Algorithms for Intelligent Crack Detection

Accurately and efficiently extracting crack information from the massive visual data collected by robots is the cognitive core that bridges the gap between physical perception and structural condition assessment. However, this visual data is seldom perfect; its quality is often compromised by physical factors such as sensor type, lighting variations, shooting distance, and angle. Consequently, the robustness of the cognitive algorithm, its ability to perform reliably on non-ideal, noisy, real-world data, is a critical measure of its engineering value. The technological evolution in this field has seen a clear shift from traditional image processing, which relies on manually designed features, to data-driven deep learning methods [52]. Due to their fixed feature extractors, traditional methods exhibit poor robustness and generalization capabilities when faced with the complex and variable scenes of the real world, thus failing to meet the demands of automated applications [53].

Deep learning, particularly Convolutional Neural Networks (CNNs), has become the mainstream technology in this field owing to its powerful capacity for automated feature learning [54]. In the specific context of robotic crack detection, the evolution of these algorithms is primarily driven by a fundamental trade-off: the need for efficiency in large-scale inspections versus the demand for precision in detailed damage quantification. This trade-off has led to two parallel yet complementary technical paths: one focusing on object detection for rapid defect localization, and the other on semantic segmentation for pixel-level contour extraction and analysis. This section provides a systematic review of these two mainstream technical approaches and the lightweighting strategies necessary for their eventual onboard deployment.

### 3.1. Object Detection Algorithms for Rapid Localization

In the context of large-scale infrastructure inspection, object detection serves as a high-efficiency triage mechanism, rapidly identifying potential crack regions with bounding boxes to focus subsequent analysis [55]. While established general-purpose detectors like the YOLO series [56], Faster R-CNN [57], and DETR [58,59] offer a solid foundation, they often struggle when faced with the atypical nature of cracks. Unlike standard objects, cracks present unique geometric challenges due to their slender, tortuous shapes and their appearance across a wide spectrum of scales and complex backgrounds. This has motivated a significant body of research aimed at adapting and refining these baseline architectures.

Early researchers focused on the most fundamental component, the convolution kernel itself, with the goal of imbuing the network with a better intrinsic understanding of non-rigid, linear features. For instance, Bai et al. [60] demonstrated that integrating a Dynamic Snake Convolution into YOLOv8 could significantly improve its ability to trace irregular curves. Concurrently, other work sought to expand the network’s receptive field to better grasp the global structure of elongated cracks, such as the Shift-Wise convolution in SCD-YOLO [61]. Given the scale variance of cracks in detection scenes, some research has focused on enhancing the multi-scale capabilities of the backbone network itself, for example, by integrating Res2Net concepts into the C3 module of YOLOv5 [62]. At the same time, a great deal of work has been devoted to designing more complex feature fusion necks, from the MsCGA attention module [63] to more elaborate network designs like GLMANet [57], all aimed at generating more robust, scale-invariant feature maps.

As capabilities for handling geometry and scale improved, distinguishing faint cracks from visually similar background clutter (e.g., stains, joints) became the next critical bottleneck. To this end, researchers began integrating various attention mechanisms to help models learn to focus on the most salient features, with mechanisms like Shuffle Attention [64] and GCSA [65] becoming common additions. Pushing this concept further, some studies leveraged the global context modeling power of Transformer modules to improve robustness against complex background interference [66]. For particularly challenging inspection environments, such as poorly lit tunnels, Duan et al. [67] developed specialized modules like semantic context encoding (SCE) and detail-preserving encoding (DPE).

Beyond architectural optimizations, advancements have also been made in the model learning process itself. At the loss function level, techniques like Focal Loss have been adopted to better handle the significant class imbalance between crack pixels and background pixels [68]. Specialized Intersection over Union (IoU) loss variants, such as WIoU and Foclar-MPDIoU [59,69], have been designed to improve the bounding box regression accuracy for highly irregular crack shapes. Research has also targeted the data itself. To mitigate the high cost and effort of manual annotation, generative models like DCGANs have been employed to synthesize diverse and realistic crack samples, providing a powerful tool for training data augmentation [70].

### 3.2. Semantic Segmentation Algorithms for Precise Quantification

Unlike object detection models that prioritize localization efficiency, semantic segmentation algorithms enable pixel-level classification, assigning a label to each pixel (e.g., crack or background) for fine-grained structural analysis [71]. This detailed output forms the basis for quantitatively measuring crack properties such as length and width, which are vital for condition assessment and structural health monitoring. The encoder–decoder framework, as popularized by U-Net [72], remains a foundational design, yet its down-sampling operations often compromise boundary precision. This limitation has prompted continued research into enhancing both local detail preservation and global structural awareness.

To address the compromised boundary precision inherent in encoder–decoder designs, one study introduces the Semantic Enhancement Refinement (SER) module, designed to strengthen the fusion of low-level textures with high-level semantic cues, thereby directly improving boundary delineation [73]. Complementary to enhancing data flow, Fang et al. [74] explored convolution techniques (DSConv) and attention mechanisms (ECA) to help the network precisely model the intricate shapes of curved and fragmented crack geometries at a pixel level, thus preventing the loss of detail during segmentation. In parallel, considering the challenges of adverse environments, some work has integrated frequency-domain transformations, such as the Discrete Cosine Transform (DCT), to mitigate poor lighting conditions [75].

While the previous refinements improve local discrimination, their limited receptive fields can lead to fragmented predictions for elongated cracks. To ensure topological consistency, Transformer-based architectures have become increasingly prominent. In segmentation, their ability to model long-range dependencies is crucial for maintaining the topological consistency of a crack mask, ensuring it is rendered as a continuous whole rather than disconnected segments [70]. This has led to hybrid models like Swin-Unet, blends with CNN backbones [71], and the adoption of efficient state-space models like Visual Mamba [72]. On a related note, some researchers have approached this connectivity challenge from a mathematical perspective, using computational topology and persistent homology to explicitly enforce crack connectivity during segmentation [73].

Beyond semantic segmentation, instance-level parsing distinguishes between individual cracks. This requires not only segmenting pixels but also grouping them into distinct instances—a key challenge when cracks intersect. To address this, specialized modules like Cascaded Group Attention (CGA) have been used to help models learn instance-specific features [76]. Concurrently, boundary-aware loss functions specifically refine the edges of each predicted instance [77].

To alleviate the reliance on large-scale, pixel-level annotated datasets, researchers are actively exploring new learning paradigms. Weakly supervised learning uses image-level labels to generate pseudo-labels for segmentation [78]. A more cutting-edge trend involves leveraging the capabilities of Foundation Models. Fine-tuning a general-purpose Segment Anything Model (SAM) on a crack dataset [79] or using the powerful zero-shot recognition capabilities of a vision-language model like CLIP to guide segmentation through text prompts [80] provides new solutions to the problem of data scarcity. To provide a clearer overview, Table 3 summarizes the core optimization techniques discussed in both object detection and semantic segmentation.

### 3.3. Lightweight Models for Onboard Deployment

Deploying deep learning models onto robot payloads with strict constraints on computing, storage, and power (e.g., UAVs [81,82,83]) or edge devices [84] is a critical step connecting algorithmic research to field applications. Standard, high-performance models are often too computationally expensive for real-time onboard processing. This has made model lightweighting a crucial research direction, focused on finding the optimal balance between inference speed (FPS), model size, and accuracy [85].

One of the main approaches to lightweighting lies in architectural redesign. This involves replacing computationally expensive components with more efficient alternatives. For instance, MobileNet employs depthwise separable convolutions to significantly reduce parameter counts [86], while GhostNet introduces a feature reuse mechanism to achieve further reductions in redundancy [87,88]. Inspired by these ideas, several crack-specific lightweight networks have been proposed. GTRS-Net introduces the GTF module to enhance encoder–decoder efficiency [87]; LMED-Net focuses on lightweight multi-scale extraction [86]; and LiteFusionNet integrates a compact spatial pyramid pooling design to preserve context at minimal computational cost [89]. Other research has involved creating lightweight variants of efficient detectors like the YOLO series. For example, Qing et al. [90] balanced speed and accuracy by introducing efficient convolution units like StarConv and a lightweight LSH detection head.

The global modeling capabilities of Transformers and state-space models (SSMs) like Mamba, previously discussed for enhancing performance, have also been integrated into lightweight architectures. Transformer-based modules, while originally heavy, are now selectively embedded into lightweight CNN backbones like MobileNetV3 [91] or paired with efficient branches such as super-resolution modules [92]. More recently, Mamba’s linear complexity has made it a core component in ultra-lightweight segmentation networks like MambaU-Light [93] and GLoU-MiT [83], and it has even been used as a standalone architecture [94]. Additionally, Spiking Neural Networks (SNNs) have emerged as a novel paradigm by leveraging event-driven computing for ultra-low-power crack detection tasks [95].

Beyond structural innovation, model compression techniques offer a route to lightweighting by post-processing existing networks. It focuses on reducing the size of an existing powerful model rather than designing a new one. Knowledge Distillation is a mainstream technique in this vein, where a compact “student model” is trained to mimic the output of a larger, more powerful “teacher model” [96]. Researchers have explored numerous distillation schemes to improve this knowledge transfer, including using multiple teachers [94] or designing instance-aware distillation methods [97,98]. This is often used in concert with network pruning, a technique that systematically removes redundant parameters from a trained network. A combined strategy of distillation and pruning has proven highly effective for achieving extreme model compression with minimal accuracy loss [99,100]. To provide a tangible comparison of these techniques, Table 4 summarizes key performance data from several representative lightweight models.

These algorithms collectively form the system’s cognitive engine, constantly navigating the trade-off between inspection efficiency and precision. An algorithm’s practical utility, however, is determined not only by its accuracy but also by its computational complexity. This complexity directly dictates its deployment strategy, a central theme in achieving the autonomous action discussed in the next section.

To provide a high-level, structured overview of the methods discussed in this section, Table 5 summarizes the main algorithmic approaches, comparing them on unified benchmarks such as performance, speed, and deployment readiness.

## 4. Autonomous Action

**Figure 3:**
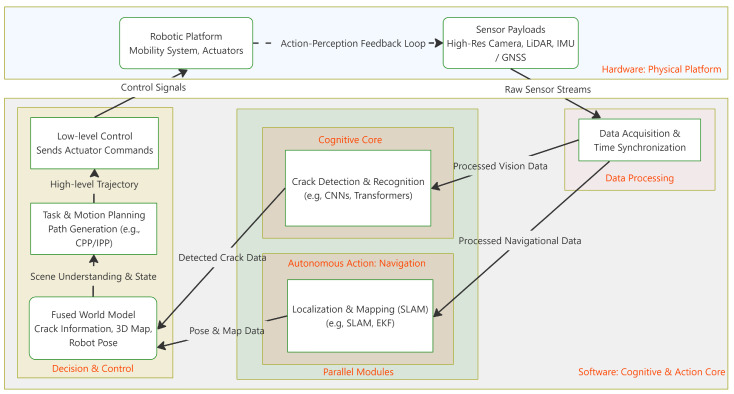
A detailed schematic illustrating the system architecture and operational flow, showing the interaction between hardware components and software modules.

The preceding sections have established the foundational modules of a robotic inspection system: the physical platform (Section 2) and the cognitive core (Section 3). This section addresses Autonomous Action, the critical process of integrating these modules to enable the leap from passive perception to active, purposeful behavior. This integration is essential for balancing the pursuit of higher autonomy with the need for on-site reliability. To provide a clearer picture of these interactions, Figure 3 presents a detailed schematic of a typical system’s architecture. It illustrates the data flow and control loops that connect hardware (the physical platform and its sensors) with the core software modules. This integrated view shows how raw sensor data is acquired and synchronized, then fed into parallel processes for cognition and action. The cognitive core uses this data for crack recognition and quantification, while the autonomous action module uses it for navigation via SLAM. The outputs from both, identified cracks and robot pose, are fused in the planning stage, which generates control commands for the robotic platform, thus closing the perception–action loop. Following this integrated workflow, this section will deconstruct the core technologies that enable autonomous action: algorithm deployment (Section 4.1), autonomous navigation and localization (Section 4.2), and high-level task planning (Section 4.3).

### 4.1. Onboard Deployment vs. Offline Processing of Algorithms

Algorithm deployment is the engineering process of running the cognitive models from the previous section efficiently and reliably on the limited resources of a physical platform. It is an indispensable link between algorithmic theory and engineering application [105], representing a trade-off among algorithm capabilities, system resources, and task requirements. This trade-off has led to two dominant deployment strategies: onboard real-time processing (edge computing) and offline post-processing.

Onboard real-time processing aims to equip the robot platform with real-time perception and decision-making capabilities, which are essential for autonomous navigation and active exploration tasks that demand immediate feedback. However, deploying models on embedded edge computing platforms, such as the NVIDIA Jetson series (e.g., Nano, TX2, Xavier NX, Orin) [106,107], presents significant challenges due to their limited computational power, memory, and energy budgets. The primary prerequisite for such deployment is the model lightweighting technology detailed in Section 3. Studies have shown that through architectural innovation and model compression, it is possible to reduce model parameters to below 1M (e.g., MDCCM [108]) or achieve inference speeds exceeding 200 FPS while maintaining high accuracy (e.g., CS-YOLO [90]), thus laying a solid algorithmic foundation for real-time onboard processing [109]. However, lightweight design alone is often insufficient; hardware–software co-optimization using inference acceleration frameworks like TensorRT (NVIDIA Corporation, Santa Clara, CA, USA) is also necessary. By applying techniques such as operator fusion, precision quantization (e.g., converting from 32-bit floating-point (FP32) to 8-bit integer (INT8)), and graph optimization, these frameworks can increase inference speeds severalfold and significantly reduce power consumption, often with only a negligible loss in model accuracy (e.g., mAP drop < 1–2%). For instance, after optimization with TensorRT on a Jetson Nano (NVIDIA Corporation, Santa Clara, CA, USA), the inference speed of the PDS-YOLO model increased from 5.4 FPS to 19.3 FPS, a performance leap that transformed the model from impractical to functional for real-time use [110].

Despite these advances, practical deployment is fraught with limitations and potential failure cases. For example, an accelerated model that runs smoothly in real-time on a high-end edge device may still fail to achieve the required performance (>30 FPS) on a less powerful one, causing the system to "drop frames" and miss critical defects during rapid inspection. Furthermore, while precision quantization from FP32 to INT8 often results in only a minor drop in overall performance metrics like mean Average Precision (mAP), this can conceal a significant performance degradation on specific, hard-to-detect samples, such as very fine cracks. Such failure cases are often hidden within broad average scores. Another critical but often overlooked limitation is the issue of thermal throttling. Under continuous high-load operation in field conditions, edge devices can overheat, forcing them to reduce clock speeds. A model that performs well in short laboratory tests might see its inference speed halve after an hour of sustained use in a warm environment, compromising the mission’s viability. These examples underscore that successful deployment requires a holistic approach that considers not just the algorithm, but also the specific hardware constraints and the harsh realities of field operation. For even more resource-constrained microcontroller applications, TinyML frameworks (e.g., TensorFlow Lite v2.9.1, Google Inc., Mountain View, CA, USA) offer the possibility of deploying intelligent algorithms on extremely low-power devices [111]. The quantitative evaluation of deployment performance typically includes core metrics such as inference speed (FPS), model size (MB), power consumption (W), and accuracy loss (%).

In contrast, offline post-processing is a decoupled strategy where the robot functions solely as a high-quality data acquisition terminal, offloading computationally intensive tasks to resource-rich ground workstations or cloud servers. This approach is advantageous when the task demands maximum detection accuracy but is less sensitive to real-time constraints (e.g., detailed damage assessment or post-disaster forensic investigations). Analyzing computationally intensive, pixel-level crack segmentation models [76] or using more complex detection models (e.g., Faster R-CNN [112] or DINO [113]) to pursue higher accuracy are both better suited to offline processing.

In practice, the final deployment solution is often a hybrid strategy tailored to specific task requirements. For example, a system might run a lightweight object detection model (>30 FPS) on the onboard device for rapid anomaly detection and navigation assistance, while simultaneously storing original high-resolution images for subsequent, detailed offline analysis. This strategic trade-off between onboard real-time efficiency and offline analysis accuracy is the core engineering decision in the algorithm deployment phase.

### 4.2. Autonomous Navigation and Long-Term Localization

A reliable localization capability is foundational to creating high-precision crack maps. For a single inspection mission, modern Simultaneous Localization and Mapping (SLAM) technology aims to ensure the local accuracy and robustness of the trajectory through tightly-coupled multi-sensor fusion. To advance this field, researchers have constructed various general-purpose multi-modal datasets, such as S3E [114] and FusionPortableV2 [115], which provide benchmarks for developing more robust SLAM algorithms. Building on this, modern SLAM frameworks tightly fuse heterogeneous sensor data through optimization algorithms like factor graphs. For example, tightly-coupled LiDAR, inertial, and camera data (LIC-Fusion) [25] can effectively suppress the drift of visual SLAM in weakly textured areas [116] and ensure the scale consistency of the map [117], thereby achieving centimeter-level localization accuracy. The performance of SLAM systems is typically quantified using metrics such as Absolute Trajectory Error (ATE) and Relative Pose Error (RPE), with high-precision systems capable of achieving an ATE of less than 5 cm.

However, for infrastructure requiring long-term, repetitive inspections, the navigation system faces a more significant challenge: balancing the need for long-term global consistency with adaptation to dynamic environmental changes. From a crack detection perspective, the practical implications of this challenge are profound. The value of a high-precision SLAM system is not merely to guide the robot’s movement but, more importantly, to assign a centimeter-level global 3D coordinate to every crack identified by the cognitive core. Without this spatial tag, the crack data loses its engineering utility. Furthermore, in long-term monitoring, the robot must be able to accurately recognize its previous location when returning to a site months or even years later. This is crucial for tracking the evolution of a specific crack and assessing the degradation trend of the structure’s health. This leads to two central problems in long-term SLAM research:

- Map Dynamism: Real-world scenes are filled with dynamic elements such as pedestrians and vehicles, which can be mistakenly incorporated as static landmarks into the SLAM system. This can corrupt the map and cause subsequent localization failures. To address this, a key strategy is to use semantic information to differentiate between static and dynamic elements. For instance, some approaches integrate semantic segmentation networks to explicitly identify and filter out transient objects like people or vehicles in real-time during the mapping process [118]. This ensures that the map is constructed using only reliable, long-term features of the environment, which is critical for maintaining localization accuracy in busy settings like human–robot collaborative manufacturing [119].
- Appearance Variability: Even a static scene can undergo dramatic appearance changes due to variations in lighting, weather, and seasons. These changes can severely degrade the performance of vision-based place recognition [120], potentially causing the Relocalization Success Rate to drop by over 50% between different seasons. This place recognition problem is a core focus of long-term SLAM research. To cope with appearance changes, a key trend is the construction of semantic maps, which are less sensitive to lighting and viewpoint. Instead of relying on unstable low-level visual features (e.g., corners, textures) that change with environmental conditions, semantic maps use higher-level, more stable objects as long-term anchors. By identifying object categories, the robot can recognize a location based on the presence and arrangement of semantic landmarks—for example, by matching a description like “a pillar is to the left of a fire hydrant” rather than raw pixel patterns. This semantic-level understanding remains consistent across different seasons or times of day, making place recognition far more robust and reliable for long-term deployments [120]. Furthermore, in complex and occluded environments like dense forests, semantic scene completion techniques can be used to predict the full geometry of the environment from partial observations, generating a more complete map that improves navigation safety and efficiency [121].

**Figure 4:**
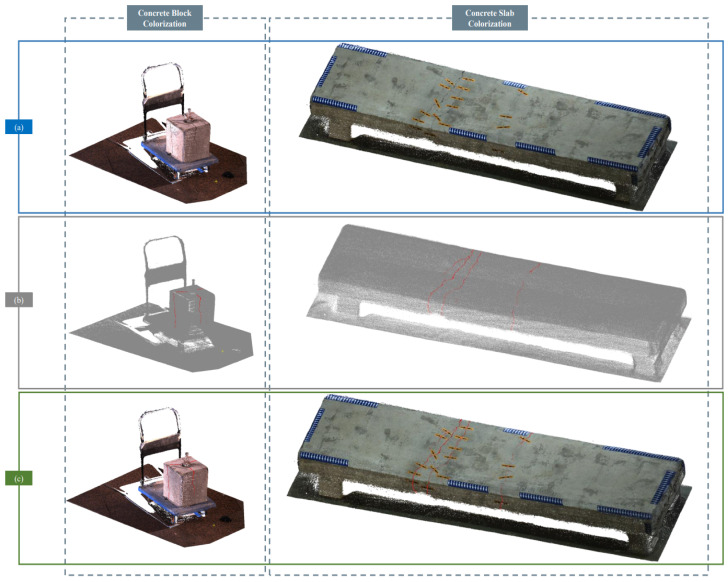
SLAM-based 3D reconstruction and crack localization [122]. (**a**) 3D point cloud generation using LiDAR SLAM; (**b**) Fusion of color data from visual camera to produce a textured 3D model; (**c**) Overlay of crack detection results onto the 3D model with global spatial coordinates.

The focus of autonomous navigation technology is thus shifting from pursuing local trajectory accuracy for single missions to addressing the global consistency challenges of long-term deployments. This evolution requires future navigation systems to be more than geometric construction tools; they must become robust spatial cognitive systems capable of understanding and adapting to changing scenes. A key enabler for this is the deep fusion of multi-modal sensor data. As illustrated in Figure 4 sourced from [122], this process transforms raw sensor inputs into actionable, spatially-tagged information. The workflow typically involves: (a) generating a 3D point cloud of the environment using LiDAR SLAM, which provides the foundational geometric map; (b) capturing color information with a visual camera and fusing it with the point cloud to create a realistic, textured 3D model; and (c) overlaying the results from the crack detection algorithm onto this model. This final step allows for the precise localization of each crack in 3D space, assigning a global coordinate to every identified defect.

### 4.3. Task Planning and Path Decision-Making

Once a robot acquires reliable navigation capabilities, it must address a higher-level question: “Where should I go next to complete the mission most effectively?” This introduces the core issue of task planning, whose fundamental goal is to balance the competing demands of ensuring complete crack detection coverage and pursuing maximum inspection process efficiency.

Coverage Path Planning (CPP) is a technical approach that prioritizes completeness. Its objective is to design a path that allows the robot’s sensor field of view to cover all areas to be inspected, thereby ensuring no potential cracks are missed and achieving a coverage rate of nearly 100% [123]. In known environments, this typically involves generating traversal paths, such as boustrophedon or spiral patterns, offline based on Building Information Modeling (BIM) or Computer-Aided Design (CAD) models. The reinforcement learning-based coverage planning model proposed by Lakshmanan et al. [124], though originally designed for cleaning robots, offers a framework equally applicable to guiding inspection robots in complete crack scanning of complex surfaces. In unknown environments, robots often adopt a frontier-based exploration strategy, continuously moving toward the boundary between known and unknown space until the entire area is surveyed. While CPP provides the strongest deterministic guarantee (e.g., >99.9% coverage), its one-size-fits-all strategy can lead to the robot expending significant mission time and energy on healthy surfaces [125].

To overcome the efficiency limitations of CPP, Informative Path Planning (IPP), or Active Perception, has emerged as a strategy that prioritizes efficiency [114]. Its core idea represents a fundamental shift: the robot’s actions should no longer be aimed at covering all paths, but at maximizing the acquisition of crack-related information [126]. In this framework, the robot acts as an active learner, evaluating the information value of different actions at each decision point and selecting the plan with the highest expected value. Here, information value is quantified by metrics such as the probability of discovering a new crack or the ability to capture clearer images of known cracks for precise width measurement. The evaluation metric thus shifts from a simple coverage rate to the improvement of the information gain rate (e.g., the number of cracks found per unit of time) within a limited mission time or energy budget. Deep Reinforcement Learning (DRL) has proven to be an effective method for training robots to learn complex reactive strategies [127,128], offering a viable path for developing next-generation intelligent inspection systems that can autonomously find and focus on potential crack areas. This transition from passive coverage to active exploration is a key step toward true robotic intelligence, but it also introduces a reliance on the accuracy of information value modeling and a corresponding risk of missed detections.

In practical applications, planning algorithms must also consider more complex real-world factors, such as the risk of collision with dynamic obstacles [129], energy efficiency during navigation [125], and social awareness in human–robot co-existing environments [130]. The most effective crack detection strategies will likely involve a hybrid application of CPP and IPP: using IPP for rapid, wide-area searches to locate potential points of interest, then initiating CPP in these local areas for detailed and complete inspection.

### 4.4. Analysis of Integrated System Case Studies

The platform, algorithms, and autonomous action strategies discussed in the preceding sections must be unified within an integrated system to create a robot with practical operational capabilities. To provide a comparative overview, Table 6 presents and compares three representative system profiles, each optimized for a distinct operational goal: high-efficiency survey, high-precision inspection, or collaborative operation. Following this high-level comparison, the section further illustrates the “Physical Platform-Cognitive Core-Autonomous Action” framework (Figure 1) by analyzing a series of recent case studies. These cases demonstrate, through clear workflows, how various technical modules are organically assembled to solve specific real-world challenges.

Case Study 1: End-to-End Autonomous Inspection for Challenging Terrains

To address the challenge of fully autonomous and efficient crack detection on winding mountain roads with weak GNSS signals, Chen et al. [131] constructed a highly integrated UAV inspection solution that aligns with the analytical framework of this study. For the physical platform, a lightweight UAV was employed for flexible maneuverability and rapid coverage. For the cognitive core, a lightweight object detection algorithm, MRC-YOLOv8, was deployed on the onboard system for real-time identification of road surface cracks. In autonomous action, at the task planning level, the system used the Sliding Window Method (SWM) to automatically generate a flight path that closely followed the road’s alignment. At the autonomous navigation level, a multi-sensor fusion algorithm provided real-time localization to compensate for GNSS drift. This solution, through its deep integration of planning, navigation, and detection, achieves end-to-end autonomous detection, serving as a model for translating theory into practice.

Case Study 2: Advanced Planning and Control for Mission-Oriented Tasks

When the mission objective is upgraded from mere detection to detection and repair, a higher level of intelligence is required. To address this, Veeraraghavan et al. [132] designed a novel "Simultaneous Inspection, Filling, and Coverage (SIFC)" method, whose workflow reflects the deep coupling of cognition and action. Here, the robot (physical platform) scans the workspace, while its onboard sensors (cognitive core) incrementally build a crack distribution map online. Then, in the autonomous action stage, the SIFC planning algorithm generates an optimal path in real-time based on the updated map. This path ensures both full sensor coverage for scanning and complete tool coverage (e.g., a filling nozzle) for all identified cracks. This case vividly illustrates that as mission complexity increases, the bottleneck shifts from the accuracy of a single algorithm to the intelligence of the task planner. The robot is no longer a tool executing a fixed path but an intelligent agent dynamically planning its behavior based on real-time perception.

Case Study 3: Integrated "Sensing–Navigation–Synchronization" for High-Precision Localization

Any collected data loses its engineering value if it cannot be tagged with precise spatiotemporal information. To achieve high-precision eddy current testing of micro-cracks in metal components, Lyu et al. [133] constructed a deeply integrated solution whose success hinges on solving the challenge of data synchronization. The solution’s workflow involves two parallel data streams: a defect signal stream (from the cognitive core) collected at high speed by an FPGA-based (Field-Programmable Gate Array) eddy current sensor, and a robot pose stream (from the autonomous action navigation module) output by the Robot Operating System (ROS) after multi-source fusion. The essence of this solution is a highly efficient software synchronization strategy that tightly couples these two heterogeneous data streams with minimal latency. The final result is that every detected defect signal can be assigned a centimeter-level coordinate in 3D space, thus achieving the leap from finding defects to precisely locating them.

Case Study 4: Comprehensive Assessment from “Crack Recognition” to “Decision Support”

Simple crack detection results offer limited utility to road maintenance authorities. Therefore, a key challenge is transforming raw data into engineering indicators that can guide maintenance decisions. Chen et al. [112] proposed a complete detection-evaluation process to address this, with a workflow covering the entire path from data to decision. First, a UAV (physical platform) collects high-definition road surface images according to a preset strategy. Second, at the cognitive core level, multiple mainstream detection algorithms (Faster-RCNN, YOLO series, etc.) are trained and evaluated in parallel. After detecting cracks, key geometric parameters such as their number, length, and area are further automatically calculated. Finally, a quantitative report on the road damage condition is generated. This case clearly demonstrates the evolution of autonomous inspection solutions from mere “data collectors” to "problem solvers", with their value lying in the successful translation of machine vision results into an actionable engineering language.

## 5. Challenges and Future Outlook

**Figure 5:**
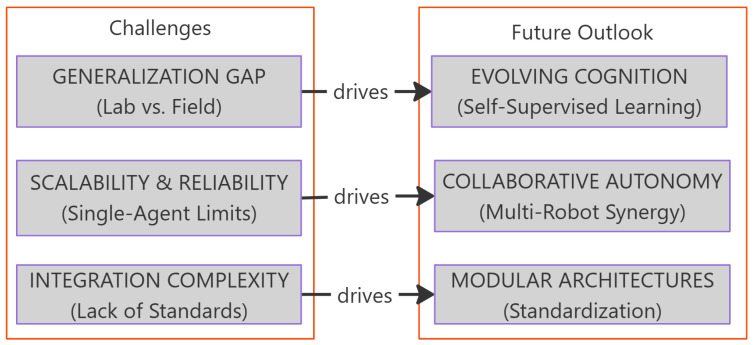
The core thesis presented in this research. The logical correspondence between the core challenges for field application (**left**) and the future solutions driven by integrated and collaborative approaches (**right**).

The preceding sections have analyzed the three core modules of a robotic inspection system: the physical platform, the cognitive core, and autonomous action. However, when this sophisticated technological system transitions from the laboratory to large-scale field applications, the inherent limitations of each module are amplified by real-world complexities, creating the core challenges that currently face the field. A deep understanding of the roots of these challenges is the logical starting point for discerning future research directions. This section examines these challenges through the lens of existing literature and looks toward future technological trends, as illustrated in Figure 5.

### 5.1. Key Technical Challenges and Application Bottlenecks

(1) Environmental Perception and Adaptability

This challenge arises from two primary sources. First, the cognitive core algorithms (Section 3) often lack sufficient generalization ability when deployed in uncontrolled, real-world environments. Second, the quality of data collected by the physical platform sensors (Section 2) can deteriorate significantly under adverse conditions. The validation of many advanced algorithms still relies heavily on datasets collected under relatively ideal conditions, such as consistent lighting and distance [73,79]. However, real-world inspection sites, such as the backlit underside of a bridge, the damp inner walls of a tunnel, or industrial pipelines with numerous stains and complex textures, are rife with environmental variables. This discrepancy causes a sharp decline in the performance of laboratory-validated algorithms in field applications, creating a significant generalization gap. For example, even state-of-the-art segmentation models may misidentify rust stains, water marks, or even shadows from vegetation as cracks, leading to high false-positive rates. Conversely, under harsh lighting conditions, fine cracks can be easily missed. Furthermore, the performance of the cognitive core is inextricably linked to the physical platform. Motion blur caused by the vibration of a UAV in windy conditions or a ground robot on uneven terrain directly degrades the quality of input images, rendering even the most advanced algorithms ineffective. These failure cases highlight that algorithmic robustness cannot be considered in isolation from its physical embodiment. As analyzed in Section 4, even advanced SLAM algorithms are prone to substantial localization drift or tracking failure in dynamically changing scenes with dramatic lighting variations, often due to feature matching failures or map corruption [118,119]. Bridging this performance gap is the central issue determining whether this technology can be truly implemented at scale.

(2) Long-term Autonomy and Reliability

This challenge highlights the inherent deficiencies of single-agent robots in terms of both efficiency and long-term reliability. On one hand, when inspecting large structures like long-span bridges or extensive tunnel networks, relying on a single robot for full-coverage inspection remains prohibitively time-consuming and fails to meet the engineering demand for rapid assessment [125]. On the other hand, as discussed in Section 4.2, ensuring that a robot can achieve reliable relocalization across multiple, long-term inspection tasks in the face of scene changes is critical for tracking crack evolution and ensuring the long-term validity of the data. This, however, remains a major weakness of current SLAM technology. Therefore, developing a new paradigm that can overcome the efficiency bottleneck of single-agent systems and guarantee long-term operational reliability is a pressing need.

(3) System Integration Complexity and Standardization

This challenge reveals a common engineering problem in technology implementation: how to reliably integrate disparate hardware (platforms, sensors) and software (algorithms, operating systems) into a stably functioning system. While most research focuses on optimizing individual modules, system failures in practice often occur at the seams between them. This is reflected in several areas: (a) Heterogeneous hardware interfaces. Sensors from different manufacturers (e.g., cameras, LiDAR) have vastly different data formats, drivers, and physical interfaces, hindering true plug-and-play hardware integration. (b) Data synchronization difficulties. As described in Case Study 3 (Section 4.4), ensuring that multi-sensor data streams are precisely synchronized at the millisecond level is a prerequisite for high-quality data fusion (e.g., for SLAM), but this is extremely challenging to achieve in practice. (c) Software stack fragility. Although the Robot Operating System (ROS) is a common development platform, its inherent shortcomings in stability, real-time performance, and multi-robot communication pose reliability risks for deployment in demanding industrial environments.

### 5.2. Future Research Directions and Technological Trends

In response to the challenges above, future technological evolution will likely be characterized not by the separate improvement of individual components, but by a systematic evolution driven by deeper integration and co-evolution, where advances in one area amplify the capabilities of others.

(1) From Data Dependency to Autonomous Learning

To fundamentally solve the generalization problem of cognitive algorithms, the core evolutionary path will be a shift from relying on static, pre-labeled datasets to enabling autonomous learning from massive, unlabeled, real-world data. The co-evolution of data and models is key to achieving this goal. On one hand, self-supervised learning has shown great potential. By designing clever pretext tasks on unlabeled data, it forces the model to learn more general and robust visual features, thereby greatly improving its adaptability to unknown scenes. On the other hand, using generative models (e.g., Generative Adversarial Networks (GANs) or diffusion models) to synthesize highly realistic and diverse crack images [134] provides a powerful new tool for data augmentation. This synergistic interplay between data and models will make the cognitive core more resilient in complex and variable physical environments.

(2) From Single-agent Intelligence to Multi-robot Collaboration

To systematically overcome the capability and efficiency bottlenecks of single-agent robots, an important emerging research direction is the use of heterogeneous fleets composed of different types of robots to achieve multi-robot collaboration. A typical collaborative workflow might be as follows. First, a fleet of highly mobile UAVs conducts a large-scale, rapid global aerial survey and 3D modeling, using efficient onboard algorithms to quickly identify potential regions of interest. Then, based on the precise 3D coordinates of these regions, the system automatically dispatches ground or wall-climbing robots with greater payload capacity and stability to perform high-precision contact-based or close-range detailed inspections. This “air-ground integrated, coarse-to-fine” model synergizes the high efficiency of aerial platforms with the detailed inspection capabilities of ground/contact-based platforms. This approach is key to achieving long-term, reliable, and efficient inspection of large structures by constructing a collective physical platform with coordinated autonomous action.

(3) Towards Modularization and Standardization

To address system integration challenges, the future research focus will likely shift from pursuing the ultimate performance of a single module to building more robust and flexible system architectures. The core of this effort is to improve the standardization and composability of system modules, ensuring that different software and hardware components can be easily replaced and integrated. At the hardware level, industry-wide promotion of standardized sensor and actuator interfaces is needed to achieve true plug-and-play capability. At the software level, the application of containerization technology (e.g., Docker) can package algorithms and their complex dependencies into independent, portable modules, greatly simplifying the deployment process across different platforms. At the same time, the new generation of robot middleware represented by ROS 2, with its native support for real-time control, Quality of Service (QoS), and multi-robot communication, is laying a solid foundation for building more stable and reliable field robot systems. This marks a leap in the value of robotic inspection systems from providing a single function to delivering a reliable service, a crucial step in the transition from technology to widespread application.

## 6. Conclusions

This study has presented a synergistic review of robotic autonomous crack detection, structured around an integrative “Platform–Cognition–Action” framework. It posits that the success of an autonomous inspection system is not defined by the performance of any single technology, but by the tightly coupled integration and synergy among the physical robot, its cognitive algorithms, and its capacity for autonomous action.

Throughout this study, we have systematically analyzed the critical trade-offs inherent at each level of the framework, supported by quantitative evidence from the literature. At the Physical Platform level, the choice involves balancing the high mobility of UAVs for rapid, large-area surveys against the stability and payload capacity of contact-based robots needed for deploying heavy NDT sensors. At the Cognitive Core, a central conflict exists between efficiency and precision. While lightweight object detectors like the YOLO series can achieve real-time speeds (e.g., 200–300 FPS) on edge devices with model sizes under 2M parameters for initial screening, more complex segmentation models are essential for the pixel-level analysis required to quantify critical crack parameters like width (e.g., resolving 0.3 mm cracks). Autonomous action represents the leap from passive perception to active decision-making, enabled by a series of complex strategies in deployment, navigation, and planning. For example, using model compression and hardware acceleration (e.g., TensorRT) can boost inference speeds on embedded systems by over 300% with minimal accuracy loss (<2% mAP), while advanced SLAM algorithms provide the centimeter-level localization accuracy (e.g., <5 cm ATE) that assigns a global 3D coordinate to every detected flaw.

The primary advantage of this framework is its practical utility as a decision-making tool. It offers more than a catalog of technologies; it provides a structured mental model for system design and evaluation. For engineers, it serves as a design blueprint, clarifying how an early choice, such as selecting a specific robotic platform, creates cascading constraints on sensor selection, onboard processing power, and ultimately, the achievable level of autonomy. It compels a holistic design process that anticipates these interdependencies. For project managers and policymakers, the framework demystifies the technology and provides a clearer basis for investment and deployment strategies. It helps answer critical operational questions: Is the goal rapid screening or detailed quantitative analysis? What are the trade-offs between a high-cost, fully autonomous system and a more reliable, semi-autonomous one? By framing the discussion around the interplay of platform, cognition, and action, this study aims to foster more informed decision-making.

In closing, the deep integration of robotics and artificial intelligence is profoundly reshaping infrastructure maintenance. By focusing on the synergy between what a robot is (Platform), what it thinks (Cognition), and what it does (Action), this study provides a structured roadmap to guide future research and practice. The ultimate goal is to accelerate the transition from laboratory prototypes to robust, field-deployable systems that make the critical task of infrastructure inspection safer, more efficient, and more reliable.

## Figures and Tables

**Table 1:** Comparison of Major sensor payloads for crack detection.

Sensor Category	Key Technology	Main Function	Industrial Adoption Rate	Limitations	Representative Literature
Visual Imaging	RGB Camera	Surface texture/color imaging	High	Light-sensitive; No depth info	[1,8,22,23]
3D and Depth	RGB-D, LiDAR	3D geometry modeling; Quantification	Medium	High data load; Surface issues	[7,24,25]
NDT	Infrared, Ultrasonic	Subsurface/internal defect detection	Low	Requires contact/proximity; Slower	[6,17,26,27]
Acoustic	Acoustic Array	Localization in GPS-denied areas	Very Low	Noise-sensitive; Not for imaging	[21]

**Table 2:** Comparison of representative robotic platforms for crack detection.

Platform Category	Key Characteristics	Advantages	Disadvantages	Representative Literature
Aerial (Multi-rotor UAV)	High mobility, 3D movement	Efficient, flexible access	Short endurance, unstable	[8,10,22,50]
Climbing (Magnetic)	Magnetic adhesion on steel	High payload, stable	Steel-only, obstacle issues	[17,38,39,40,41,42]
Climbing (Negative Pressure)	Suction on smooth surfaces	Widely applicable	Needs smooth surface, high energy	[4]
Confined-Space (Pipe Robot)	Deformable/bionic structures	Access to internal areas	Low payload, navigation difficulty	[43,44,45,46,47,51]
Ground Mobile	Wheeled/legged movement	High payload and endurance	Ground-level access only	[37,48,49]

**Table 3:** Core Optimization techniques for intelligent crack detection.

Technology Category	Specific Technology/Method	Core Function and Purpose	Representative Literature
Convolution Unit Optimization	Dynamic Snake Conv	Adaptively fits kernels to non-rigid crack geometry.	[60,74]
	Shift-Wise Convolution	Expands receptive field to capture global structure.	[61]
Multi-scale Feature Fusion	Res2Net (integrated in C3)	Extracts multi-scale features at a finer granularity.	[62]
	MsCGA Module	Refines fusion of high-level features via attention.	[63]
Attention Mechanisms	Shuffle Attention	Lightweight grouped spatial and channel attention.	[64]
	GCSA/ECA	Enhances focus on key features via global/channel attention.	[65,74]
	Cascaded Group Attention (CGA)	Progressively refines features for instance segmentation.	[76]
Global Context Modeling	Swin Transformer	Hierarchical vision transformer for global modeling.	[70]
	Visual Mamba	Efficient global context modeling with linear complexity.	[72]
	Persistent Homology	Topology-aware loss to ensure segmentation connectivity.	[73]
Loss Function Optimization	Focal Loss	Focuses model on hard-to-classify samples.	[66]
	Wise-IoU (WIoU)	Improves localization accuracy for irregular targets.	[69]
	Boundary-aware Loss	Adds supervision to enhance segmentation boundary sharpness.	[77]
Advanced Learning Paradigms	Weakly/Self-supervised	Learns from image-level labels or unlabeled data.	[78]
	Foundation Models (SAM, CLIP)	Fine-tunes/prompts large models for efficient segmentation.	[79,80]

**Table 4:** Performance Comparison of representative lightweight crack detection models.

Model/Method	Platform/Target	Model Size (Params)	Inference Speed	Key Accuracy Metric	Ref.
CS-YOLO	General/Concrete	2.03 M	221.3 FPS	89.7% mAP50	[90]
CrackScopeNet	UAV Platform	1.05 M	-	82.1% mean Intersection over Union (mIoU)	[81]
LiteFusionNet	General/Road	0.493 M	3.69 ms	64.3% mIoU	[89]
YOLOv7 BiFPN-G	Edge Device	7.4 M	-	-	[101]
YOLOv8-LUAPD	UAV/Edge Board	2.646 M	33.1 FPS	71.5% mAP50	[82]
YOLO v5-DE	Mobile Device	1.4 M	295.8 FPS	>96% Accuracy	[98]
Dual Encoder Net	Portable Device	<2 M	-	87.4% F1-score	[102]
CarNet	General/Road	4.89 M	104 FPS	51.4% ODS F-score	[100]
Distillation Net	Mobile Robot/Edge	6.8 M	77.7 FPS	84.4% Precision	[97]
MambaU-Light	Resource-constrained	3.66 M	-	84.3% mIoU	[94]
Improved YOLOv8n	Underwater Robot	1.6 M	261 FPS	93.3%precision	[103]
YOLOv8-CD	Mobile Terminal	-	88 FPS	93.8% mAP50	[104]
TF-MobileNet	NVIDIA Jetson Nano	-	-	90.8% mAP50	[91]

**Table 5:** Comprehensive Comparison of Core Crack Detection Approaches.

Approach Category	Representative Method/Model [Ref]	Accuracy (Metric and Value)	Speed (FPS)	Deployment Readiness and Scenario
Traditional IP	Edge Detection/Thresholding [53]	Low (Qualitative)	N/A	Low Readiness. Unsuitable for robust field deployment; serves as a historical baseline.
Object Detection	Faster R-CNN (Two-stage) [57]	High (Baseline for accuracy)	∼10	Medium Readiness. Suitable for offline analysis or powerful edge devices where localization accuracy is prioritized.
	YOLO-series (One-stage) [56,89,98]	mean Average Precision (mAP)@0.5: 89.7–96%	>200	High Readiness. The de facto standard for real-time onboard screening on resource-constrained platforms (e.g., UAVs).
Semantic Segmentation	Swin-Unet (Transformer-based) [70]	Very High (State-of-the-art)	<20	Low Readiness (for onboard). Best for high-precision offline analysis. Critical for quantifying long, complex cracks.
	U-Net (CNN-based baseline) [67]	Good (Baseline for segmentation)	∼30	Medium Readiness. A versatile and common backbone for many segmentation tasks, often adapted for specific needs.
	CarNet (Lightweight CNN) [100]	ODS F-score: 51.4%	104	High Readiness. Enables real-time pixel-level analysis on mobile robots, trading some precision for high speed.
Instance Segmentation	YOLOv8-based (Mask-head) [76]	Mask mAP: High (Qualitative)	∼50	Medium Readiness. Required for advanced analysis, such as counting or measuring individual crack instances.
Foundation Models	Segment Anything Model (SAM) [79]	Varies (Zero-shot performance)	<5	Very Low Readiness. Currently in the research stage. Not yet practical for real-time deployment due to huge size and slow speed.

**Table 6:** Comparative overview of high-performance integrated system configurations.

Profile	Config. 1: High-Efficiency Aerial Survey	Config. 2: High-Precision Contact Inspection	Config. 3: Collaborative Air–Ground System
Representative Study	Chen et al. [131]	Dalmedico et al. [17]	Chu et al. [37]
Application Scenario	Fast inspection of roads and bridges	NDT for welds in high-temperature areas	Full inspection of complex infrastructure (e.g., tunnels)
Hardware Core	UAV + HD Camera	Climbing robot + Ultrasonic probe	Quadruped robot + UAV
Cognitive Core	Lightweight real-time detection (e.g., YOLO)	Signal processing for defect analysis	Crack segmentation (offline)
Core Advantage	High-speed, wide-area coverage	High-precision localization under harsh conditions	Synergizes ground endurance with aerial flexibility
Key Trade-off	Less precise for fine cracks; sensitive to lighting	Slower; limited to ferromagnetic surfaces	High complexity in integration and coordination

## Data Availability

No new data were created or analyzed in this study.
